# Physical activity on prescription for children with obesity: a focus group study exploring experiences in paediatric healthcare

**DOI:** 10.3389/frhs.2024.1306461

**Published:** 2024-04-04

**Authors:** Charlotte Boman, Susanne Bernhardsson, Stefan Lundqvist, Karin Melin, Katarina Lauruschkus

**Affiliations:** ^1^ Region Västra Götaland, Centre for Physical Activity, Gothenburg, Sweden; ^2^ Unit of Physiotherapy, Department of Health and Rehabilitation, Institute of Neuroscience and Physiology, Sahlgrenska Academy, University of Gothenburg, Gothenburg, Sweden; ^3^ Region Västra Götaland, Research, Education, Development and Education Primary Health Care, Gothenburg, Sweden; ^4^ Institute of Health and Care Sciences, Sahlgrenska Academy, University of Gothenburg, Gothenburg, Sweden; ^5^ Region Västra Götaland, Department of Child and Adolescent Psychiatry, Sahlgrenska University Hospital, Gothenburg, Sweden; ^6^ Department of Health Sciences, Faculty of Medicine, Lund University, Lund, Sweden; ^7^ Department of Habilitation, Committee on Psychiatry, Habilitation and Technical Aids, Malmö, Sweden

**Keywords:** children, obesity, physical activity on prescription (PAP), paediatric healthcare, focus group, determinants, implementation, Normalization Process Theory

## Abstract

**Background:**

Insufficient physical activity is a growing public health concern and is closely linked to obesity in both adults and children. Swedish physical activity on prescription (PAP) is effective in increasing physical activity levels in adults, but knowledge about how PAP is used in paediatric healthcare is lacking. Therefore, this study aimed to explore experiences of working with PAP for children with obesity amongst paediatric staff and managers.

**Methods:**

Seven focus group discussions with 26 participants from paediatric outpatient clinics in western Sweden were conducted. Data were analysed both inductively and deductively, framed by the Normalization Process Theory's four core constructs: coherence, cognitive participation, collective action, and reflexive monitoring.

**Results:**

The PAP work for children with obesity was experienced to be about helping children to become physically active, and less about losing weight. Identified barriers for using PAP were the non-uniform nature of the work and a perceived lack of guidelines. Collaboration with physiotherapists and physical activity organisers outside the organisation was identified as an important facilitator. An important contextual factor for implementing PAP is the collaboration between paediatric clinics and physical activity organisers. In the transition between these stakeholders, maintaining a family-centred approach when working with PAP was experienced as challenging.

**Conclusions:**

PAP is a well-known intervention that is inconsistently used for children with obesity. The intervention should include a family-centred approach for this patient group. It also needs to align better with existing collaborations with other healthcare units as well as with new forms of collaboration with physical activity organisers in the community.

## 1 Introduction

An increasing number of countries are experiencing high childhood obesity rates (1, 2), making this an urgent public health issue. The prevalence of obesity in European children aged 5–9 years was 11% in 2016 (3). In Sweden, 6% of children aged 6–9 suffered from obesity in 2019, a 4% increase since 2016 (4). The condition is complex and caused by multiple factors (e.g., genetic, environmental and lifestyle factors); it often remains into adulthood and is associated with cardiometabolic and psychosocial comorbidity, as well as early mortality (5–7). Following the COVID−19 pandemic, the prevalence has continued to increase even more (8), as a result of physical inactivity, excess screen time and food consumption (9). A risk factor, particularly evident in middle childhood and adolescence, is insufficient physical activity (PA) (10–12). Physical activity is a key contributor to wellness, healthy development, and the prevention of obesity in both children and adults (13). To mitigate health risks, children are recommended to engage in PA for at least 60 min per day at moderate to vigorous intensity levels (14), but a high percentage of children with obesity do not reach these recommendations (15). To date, results of efforts to increase PA amongst children with obesity have been inconsistent, with little to no effect on time spent in PA (16–18). Despite this scarcity of effective interventions, insufficient PA amongst children must be addressed.

Behaviour change interventions are often used to influence physical activity, sedentary behaviour, and dietary habits (19). One such intervention, targeting physical activity and sedentary behaviours in individuals that are insufficiently physically active, is the Swedish method “physical activity on prescription” (PAP) (20). This intervention is based on three core components: a person-centred dialogue, an individually tailored activity recommendation with a written prescription, and a structured follow-up (21). Additionally, two further components are evidence-based guidance and collaboration with activity organisers (20, 21). The Swedish PAP model has been shown to increase PA levels in adults (22), but for children, the scientific basis for PAP is insufficient. A few small studies have investigated PAP alone or as part of a combined intervention, and suggested increased PA levels, reduced age- and sex-adjusted body mass index (ISO-BMI), and increased motivation for PA, respectively (23–25).

Despite the scarcity of research, PAP has been used in Sweden for insufficiently physically active children to various extents for several years, with positive clinical experiences. Regional clinical guidelines and routines have been developed, which highlight the importance of a family-centred approach and the need for adapting the individualised counselling and written prescription for self-selected PA to the child's age and maturity (20). Licensed healthcare practitioners, e.g., physiotherapists, nurses or paediatricians, with knowledge of the PAP method, may use the intervention and write a prescription for PA. The prescribed PA can be an everyday activity, such as cycling to school, or an organised physical activity, such as playing football. As a first step before implementing a new intervention in healthcare, factors that might hinder or enable improvements in practice need to be identified (26). These factors, or implementation determinants, exist at all levels of the system including micro (i.e., individual), meso (i.e., organisational) and macro (i.e., policy) levels (27). In a recent cross-sectional study (28) investigating the perceptions of PAP for children with obesity in paediatric healthcare, staff and managers reported the intervention to be familiar, by many viewed as a normal part of routine practice, and widely accepted for children with obesity. Main reported barriers were inadequacies of education, resources and research on PAP for children, while facilitators were understanding PAP and its components and taking on the role of using it.

To enhance understanding of how PAP is used for children with obesity in paediatric healthcare, barriers and facilitators for implementing PAP in this context need to be further explored using a qualitative research approach. This has been done previously in adult populations (29–31), in paediatric healthcare for children with intellectual disability (32), and in a school context (25). To our knowledge, no study has explored determinants for implementing PAP for children with obesity in paediatric healthcare. Therefore, the aims of this study were to explore (1) experiences of working with PAP for children with obesity amongst staff and managers in paediatric clinics; (2) perceived barriers and facilitators related to implementing PAP for children with obesity; and (3) contextual factors considered important for working with PAP for children with obesity.

## 2 Materials and methods

### 2.1 Study design

This is an explorative study with a qualitative design using data from semi-structured focus group discussions. Focus groups were chosen because they offer a more natural setting for participants to be influenced by each other, almost as in real life, than individual interviews (33). In focus group discussions, the participants can modify their experiences through interaction with others, which leads to the generation of new knowledge, according to a social constructivist approach (34). They are also suitable for uncovering the range of perceptions that people have about a topic and highlight factors that influence their opinions, behaviour, or motivation as well as the expressions and wording they use (33). The study combines an inductive and deductive approach, applying an implementation framework. It builds on and deepens the findings from the previously conducted cross-sectional survey (28), both of which are part of a larger research project about implementation of PAP for children with obesity in paediatric healthcare (35).

The study is reported according to the Consolidated criteria for reporting qualitative research (COREQ) guidelines for qualitative studies (36) (Supplementary File S1).

### 2.2 Theoretical framework

Exploring prerequisites for implementing an intervention is crucial for implementation success. In the complex healthcare context (37), working with implementation may be challenging. To understand the implementation process, using a theory is recommended (38, 39). Normalization process theory (NPT) is an implementation theory that is frequently used in qualitative research to explain processes that shape the translation of interventions in organisations and delivery of healthcare (40). The theory aims to explain the work people do during the implementation process and comprises four core constructs: coherence (sense-making work of a new practice); cognitive participation (relational work to build and sustain a community of practice around a new intervention); collective action (operational work to enact a new practice); and reflexive monitoring (appraisal work to understand the new practice) (41). The constructs motivate and shape implementation processes, by focusing on how an intervention becomes feasible and integrated into clinical practice. The NPT and its core constructs informed the developing of a coding framework, used in our analysis to gain an understanding of the experiences of staff and managers of working with PAP. This framework also offers the possibility of using sub-constructs to support interpretation at a more detailed level (40).

### 2.3 Study context

The study was conducted in the specialised paediatric healthcare organisations in Region Västra Götaland, which is Sweden's second largest county council. Region Västra Götaland was chosen as the study arena mainly for pragmatic reasons; the research group is based in this area. These organisations work with a holistic view on children and youth whose health and development are dependent on many surrounding factors and are tasked with providing support to children with obesity (41). Altogether, there are 26 paediatric outpatient clinics in Region Västra Götaland. In this study, 11 of those, located in Gothenburg and surrounding municipalities, were involved. Two rehabilitation clinics providing healthcare services for children with obesity in collaboration with the paediatric clinics also participated. The paediatric clinics are organised in interprofessional teams consisting of nurses, paediatricians, dieticians, psychologists, and occasionally consulting physiotherapists from rehabilitation clinics.

Children are referred to these clinics from primary healthcare and school healthcare. When working with the PAP method, healthcare practitioners explore the child's current physical activity habits, select suitable activities together with the child and their parents, and then issue a prescription for these activities. Some of the physical activities prescribed are free or discounted, and the activities are tailored to each individual child. Planning and performing or participating in the activity is the responsibility of the family, but they may receive support by the healthcare practitioner and/or the activity organiser. Examples of activity organisers include sports clubs, gyms, and other municipality-based or non-profit organisations. In Gothenburg, the delivery of PAP is supported by a specialised unit providing education, tutoring, networking, and an activity catalogue with a compilation of activities within the city. There are also PAP clinics and rehabilitation clinics supporting families whose children have been prescribed physical activity and need extra support.

### 2.4 Procedure and participants

Two senior managers in charge of the paediatric outpatient clinics and two unit managers for the rehabilitation clinics were contacted to obtain buy-in for the study and consent to recruit participants within their organisation. Information about the study was then sent by email to approximately 200 staff and managers working with children with obesity, selected with the assistance of managers and administrative staff. The inclusion criteria were to be either staff or manager at a paediatric outpatient clinic or a rehabilitation clinic tasked with treating children with obesity, and to have experience of work with PAP at the clinic for this patient group. Experience of direct, clinical work with PAP was not a requirement as we were interested in the collective experience of PAP work. The recruitment period lasted two weeks in April 2021, including two email reminders.

Thirty-one potential participants expressed their interest in participating and were contacted by the first author (CB). A purposeful sampling strategy was used, aiming to include different professions from various clinics to achieve maximum variation. When composing the groups, both homogeneity and heterogeneity were strived for (34). To achieve homogeneity, i.e., sharing a common experience, managers and staff were assigned to separate focus groups, while heterogeneity or diversity within the groups was strived for by mixing age, profession, and workplace. Five participants later declined due to lack of time, change of workplace, or for unknown reasons. Twenty-six participants (24 women and two men), aged 28–63 years, were included. Of those, 17 were staff and nine managers, divided into seven groups (Table 1). Two nurses had additional assignments as development managers, but since their main work tasks were clinical, they chose to be part of the staff groups.

**Table 1:** Overview of participants in the focus groups.

Focus groups	*N*	Gender	Age	Years of experience**^a^**	Trained in motivational interviewing	Trained in PAP
** **	** **	W/M	Years	Range	*N*	*N*
A = staff^b^, five groups	18	18/0	28–63	0–27	11	13
B = managers^c^, two groups	8	6/2	45–55	0–20	4	2

PAP, physical activity on prescription; W/M, women/men.

^a^ Years of experience in the organisation.

^b^ Assisting nurse (*n* = 1), dietician (*n* = 2), nurse (specialist nurse in paediatric or primary health care) (*n* = 7), paediatrician (*n* = 3), physiotherapist (*n* = 3), and psychologist (*n* = 2).

^c^ Unit manager, area manager, senior manager.

### 2.5 Data collection

The focus group discussions were conducted during work hours between May and August 2021. Due to COVID-19 restrictions and to save travel time, they were video-recorded using the Zoom™ platform (Zoom Video Communications Inc., San José, California, USA). All focus group discussions were conducted by CB and KL, taking turns acting as moderator and observer. Before the sessions started, the participants filled out a form with data on age, gender, geographic location, training in motivational interviewing, training in PAP, profession, and years of experience in the organisation (Tables 1, 2).

**Table 2:** Normalization Process Theory coding framework for work with physical activity on prescription.

NPT construct	Subconstruct
Coherence: How do people work together to understand and plan the activities that need to be accomplished to put PAP and its components into practice?	Differentiation: How do people distinguish PAP and its components from their current ways of working?
	Communal specification: How do people collectively agree about the purpose of PAP and its components?
	Individual specification: How do people individually understand what PAP and its components require of them?
	Internalisation: How do people construct potential value of PAP and its components for their work?
Cognitive participation: How do people work together to create networks of participation and communities of practice around PAP and its components?	Initiation: How do key individuals drive PAP and its components forward?
	Enrolment: How do people join in to work with PAP and its components?
	Legitimation: How do people agree that PAP and its components are the right thing to do and should be part of their work?
	Activation: How do people continue to support PAP and its components?
Collective action: How do people work together to enact PAP and its components?	Interactional workability: How do people do the work required by PAP and its components?
	Relational integration: How does using PAP and its components affect the confidence that people have in each other?
	Skill-set workability: How is the work with PAP and its components appropriately allocated to people?
	Contextual integration: How is the work with PAP and its components supported by host organisations?
Reflexive monitoring: How do people work together to appraise PAP and its components?	Systematisation: How do people access information about the effects of PAP and its components?
	Communal appraisal: How do people collectively assess PAP and its components as worthwhile?
	Individual appraisal: How do people individually assess PAP and its components as worthwhile?
	Reconfiguration: How do people modify their work in response to their appraisal of PAP and its components?

PAP, physical activity on prescription.

A discussion guide with five key questions was developed (Supplementary File S2), based on discussion with field professionals, literature, and the results of our previous cross-sectional study (28). The discussion guide was not pilot-tested, as it built on the answers to the open-ended questions from the previous cross-sectional study (28). As it worked well in the first focus groups, it was not revised for subsequent focus groups.

To introduce the topic, the session started with an ice-breaker question (34): “What, in your opinion, is PAP?”. The observer then briefly summarised what was said and explained the core components of PAP so that everybody would have a common understanding of the method, especially those who did not have any direct experience. The moderator then guided the discussions using the key questions and encouraged the participants to talk freely and present alternative views. Prompts such as “anyone else with the same experience?”, “tell me about your needs?”, and “can you give an example?” were frequently used. The discussions lasted between 40 and 85 min. At the end of the sessions, the moderator summarised the discussion, and the participants were invited to comment and address anything else that they wanted to add. The observer took field notes and observed the verbal and non-verbal conversation flow. The recordings were transcribed verbatim by a transcribing service, and CB verified the transcripts. The transcripts were also returned to all participants for comments and corrections.

### 2.6 Data analysis

Data were analysed using the methodology for focus groups described by Krueger and Casey (33). Both manifest and latent content were analysed. The analysis process combined an inductive, data-driven approach in a first phase with a deductive approach in a second phase, in which NPT was applied as an analytical framework.

Phase 1: The analysis process started during the debriefing meetings after each session. When all data had been collected, the authors familiarised themselves with the collective narratives by watching the Zoom sessions and reading the transcripts several times to gain a sense of the whole. All parts of the text that were related to the key questions were assigned relevant codes. The first transcript was coded independently by CB, SB, and KL, after which the coding strategy was discussed, and codes adjusted according to consensus. The remaining transcripts were coded by CB and content conformity was verified by KL. The next step was to look for patterns and similarities as well as contrasting content across the data, which were summarised descriptively and organised into subcategories and categories. The emphasis was put on the frequency and extensiveness of what was said, but also how it was described and whether it was said with emotion (33). Interpretation was facilitated by the field notes and the video recordings. Consensus on category labels and content was reached through continuous discussions amongst the authors in an iterative process, and categories were developed and structured inductively.

Phase 2: To seek further insights and potentially explain the experiences of working with PAP by applying the NPT framework, categories and subcategories were sorted deductively into the four NPT constructs (Table 2). To support a more detailed interpretation, relevant NPT subconstructs were used (distinguished by italic font in the results presentation).

The most salient findings were illustrated with quotes with essential content, with particular focus on capturing interaction amongst the participants (33). Quotes are distinguished by type of focus group (A = staff; B = managers), participant (A1–A18; B1–B8), and their profession. The Nvivo^©^ version 12 software (QSR International Pty Ltd.) was used to manage the data and support the analysis.

### 2.7 Researcher characteristics

The focus group discussions were conducted by CB, physiotherapist, and PhD student with a clinical focus on PAP and paediatric healthcare, and KL, physiotherapist, and PhD with a research focus on PAP for children. The research group also consisted of SB, physiotherapist, and associate professor with a research focus on implementation research, KM, psychiatric nurse, and PhD with a research focus on children and SL, physiotherapist, and PhD with a research focus on PAP in adult populations. All have experience in both qualitative and quantitative research in healthcare in Region Västra Götaland and KL in Region Skåne, Sweden. CB had previously engaged with six of the participants as an educator in PAP for children, conducted PAP network events, and visited workplaces to inform about PAP.

## 3 Results

The analysis resulted in eleven categories and 20 subcategories, organised under four themes relating to the NPT core constructs: Coherence: *The potential value of PAP for the child with obesity;* Cognitive participation: *Inconsistent use of PAP amongst co-workers;* Collective action: *Collaboration around PAP across settings*; and Reflexive monitoring: *New insights about PAP* (Table 3).

**Table 3:** Staff and managers’ perceptions of working with physical activity on prescription for children with obesity.

Sub-category		Category	NPT construct: theme
Increasing health and wellbeing	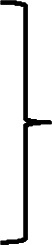	Useful to get started with PA	Coherence *The potential value of PAP for the child with obesity*
Starting facility-based PA			
Increasing everyday activity and decreasing sedentary behaviour			
** **		A tool for changing PA behaviour	
PAP is similar to usual ways of working	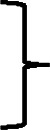	Varying perceptions of PAP and its components	
Basic understanding of PAP			
** **		An unclear mission	Cognitive participation *Inconsistent use of PAP amongst collaborators*
Nurses and paediatricians initiating and working with PAP at the clinics	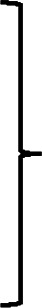	Professionals in different contexts working with PAP	
Physiotherapists missing in the teams but working with PAP outside the clinics			
PA organisers working with PAP outside the clinics			
Digitising the method	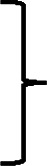	Developing and using support systems	
Supporting communication with visual aids			
Lack of collaboration with PA organisers	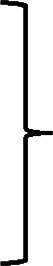	Organisers of physical activities playing a vital part	Collective action *Collaboration around PAP across settings*
PAP entitling to cost-reduced activities			
No given place in organised sports for children with obesity			
Socioeconomic conditions	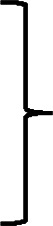	Complex family situations	
Cultural differences			
Parental conditions			
Working family-centred makes sense	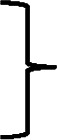	Using a family-centred approach	
Expanding to “Family PAP”			
** **		Participants reconfiguring their work after receiving information about the method	Reflexive monitoring *New insights about PAP*
Barriers for working with PAP	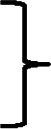	Determinants for working with PAP exist in all domains	
Facilitators for working with PAP			

NPT, Normalization Process Theory; PA, physical activity; PAP, physical activity on prescription.

The themes are somewhat related, just as there is a certain overlap between the core constructs. The varied understanding of PAP among staff and managers affects their use of the method and collaboration both with other healthcare units and with activity organisers, which is reflected in the second and third theme. The inconsistent use of PAP amongst collaborators that may hinder cognitive participation also make collaboration with different stakeholders more difficult in terms of collective action. The potential value of PAP for children with obesity identified within the first theme contributed to the new insights about PAP described in the fourth theme.

During the analysis process, it was noticed that participants regularly used a variety of inconsistent terms when referring to PAP, such as PAP-hours, PAP-station, PAP-level, PAP-center, PAP-checkpoints, PAP-resources, child-PAP, family-PAP, and PAP-dietician. While some of the participants' words were already used in clinical practice, others appeared to be spontaneous in the discussions. The numerous terms were perceived as confusing to both the participants and the researchers at times.

### 3.1 Coherence: the potential value of PAP for the child with obesity

In relation to this construct, the participants described how they manage their work with PAP for children with obesity based on their understanding of PAP. Coherence work, i.e., understanding and making sense of the intervention and its components, was required to understand the potential value of using PAP and to discern which children would need it the most.

#### 3.1.1 Useful to get started with physical activity

There was *communal specification* amongst the participants that PAP is primarily a tool to facilitate getting started with physical activity. Managers talked about it in general terms, such as getting started to improve health, have fun and be with friends. Staff expressed it more in terms of categories such as participating in organised physical activities, increasing everyday activities, and decreasing sedentary screentime.

*For me it's a tool, //…// to get families started who are not physically active by themselves. It's not a tool for families who already are physically active and already have a lot of movement and where the children might already participate in any sport. Then maybe that's not something you want to add. No, it's rather for those who sort of start from zero, that's where I have used it.* (A15, nurse)

*I think so too.* (A14, nurse)

*I agree with you both.* (A16, nurse)

Working with everyday activities and daily routines at home were closely connected to working with reducing sedentary screen time. The participants could see that parents needed such guidance and support for their children, but they also expressed that sedentary behaviour is new to them and a challenging subject.

#### 3.1.2 A tool for changing physical activity behaviour

*Individual specification* was demonstrated amongst participants' descriptions of their work to initiate PA and bring about habitual change through working with PAP. They used the method as a structured work tool and described their use of all the components. The person-centred dialogue component was seen as very important, but in general it was the least mentioned component during the focus group discussions. One participant described how the dialogue facilitated informing about body functions, such as getting tired, sweating, and increasing blood circulation during PA, which parents were perceived to have limited knowledge about. Staff also described how they used counselling to evoke the children's and parents' motivation, support daily routines and strengthen the children to become healthy grown-ups.

There was also a shared understanding that framing PAP as an intervention to influence weight should be avoided in talks with the family.

*What's the purpose of PAP? For the family, it's for the child to lose weight. That's how they think. //…// Thinking that they should lose weight is completely unreasonable. It will just be a failure all the time. You need to be very clear about why you should have PAP. It is to get you started with physical activity.* (A16, nurse)

*PAP definitively but if they're going into this to lose weight then we know it's completely unreasonable.* (A13, dietician)

Participants agreed that the method should not be used to lose weight, which they regarded as a barrier for the work with PAP, decreasing motivation for the child and instilling false hope for the families.

#### 3.1.3 Varying perceptions of PAP and its components

It was not always easy for the participants to *differentiate* PAP from their general work with PA and they considered their regular practice routine to be similar to the PAP method. Normal practice included making an agreement, setting goals, and following up, only that they did not call it PAP and did not issue a written prescription.

*I think that perhaps PAP is something we use all the time because we recommend physical activity. Writing it down… I think more about trying to organise, stimulate and plan physical activity. Can that count as PAP, that's my question?* (A17, physician)

While several participants worked with PAP and its components, others perceived that they needed more individual and common understanding of the intervention, especially the written prescription, to feel more confident about it.

### 3.2 Cognitive participation: inconsistent use of PAP amongst collaborators

In relation to this construct, participants described how they strive to work together to create networks of participation and communities of practice around PAP. Participation was experienced as largely dependent on external collaboration and less on work assigned to the unit alone. Cognitive participation was impeded due to the inconsistent use of PAP, both within and outside paediatric healthcare, which limited the participants' possibilities to drive PAP forward, expand the network of participation, and achieve sustained support for using the method.

#### 3.2.1 An unclear mission

The participants asked themselves whether there is a formal mission to facilitate the PAP work for children with obesity or not. In relation to collaborators, they perceived both availability and support to be unequal. Managers wished to overcome this barrier and called for a concept for how to work with PAP. They expressed a need for consensus around how and when PAP should be used based on a family-centred approach and equal care. Prerequisites for *initiating* the work with PAP were described as inadequate, indicating a need for more formal structures. The ambiguities around the mission, as well as the unclear definitions of aim and content of PAP, were perceived as contributing to issues about *legitimation*, including difficulties to agree that PAP is suitable as treatment for children with obesity.

*I don't think you can fix it (the PAP work) yourself in a paediatric clinic without support from the environment. It feels overwhelming.* (B7, manager)

*We don't have any physiotherapists if we start there.* (B6, manager)

*There are prerequisites for writing the prescription and the knowledge is with the staff //…// Those prerequisites do exist but if it means that we must organise the entire unit with the activity as well, then it doesn't exist. //…// We need an external partner that we can work with.* (B8, manager)

#### 3.2.2 Professionals in different contexts working with PAP

While managers expressed concerns about the formal structure, staff described their *enrolment* in PAP as non-uniform and varying from one clinic to another depending on local contexts. Nurses and physicians were perceived as key people *initiating* the intervention. Although PAP was sometimes delivered at the clinics, work with PAP more often consisted of initiation and writing the prescription, and then assigning continued work with PAP either to physiotherapists at external clinics or to organisers of physical activities. This arrangement was perceived to better suit both working conditions at the paediatric clinic and children's needs.

Managers discussed that there is a lack of physiotherapists in paediatric healthcare because there is no formal provider agreement for this profession. Nevertheless, physiotherapists external to paediatric healthcare are commonly involved in PAP work due to their skills in assessing and treating physically inactive children through tailored support. Participants described using the written prescription to refer children to physiotherapists at external clinics.

*There are two rehab places you can send them to. It should be everywhere, really.* (A23, nurse)

*We have a lot of collaboration with a rehab clinic and many PAP prescriptions are written from that clinic. They take this on, and it works very well.* (A24, psychologist)

*I have something similar, a close collaboration with paediatric physiotherapists //…//* (A22, manager)

*//…// once every two weeks the physiotherapist from rehab has their clinic with us on site, and that can help bridge the gap so that she gets the child or family to come to the rehab clinic. I think it can be an important thing that helps.* (A24, psychologist)

A few clinics collaborated with physiotherapists coming on site to see the children, which was perceived to bridge the gap for families transitioning between clinics. In these cases, the written prescription was seldom used. Writing the prescription was generally perceived as time consuming and associated with double work, because it is not integrated into the child's regular care plan but is located elsewhere in the medical chart. Often a treatment period with PAP was initiated later after gross motor assessment and/or individual treatment by physiotherapists.

Another type of collaborator that was discussed were organisers of physical activities to whom the participants referred the written prescription, to receive cost-reduced exercise for the children.

*PAP can give access to a cost reduction on certain activities.* (A13, dietician)

*If a parent goes to the gym, does the child join for free or pay a reduced price?* (A14, nurse)

*Do not know. //…// According to PAP they can possibly hang out with their parents. I dońt know what it looks like. Then there are different gyms. I work in four municipalities and there is clearly no uniformity in this either.* (A13, dietician)

Based on their experience of using PAP the participants found it challenging to stay up to date on the current range of activities being offered. Therefore, they suggested referring the child to someone with such knowledge, but they also wanted increased availability of local activity offerings and easy access through e.g., an activity catalogue, that preferably should be internet-based. Another challenge mentioned by participants who used the written prescription as a referral only was that they expected a referral response. A confusion arose because they expected a response, and when it did not come, the follow-up was also experienced as unclear and confusing.

#### 3.2.3 Developing and using support systems

Participants discussed various ideas of what could facilitate and provide continued support for the work with PAP, for example digitising the prescription or the activity diary and visual aids. Suggestions for *activation* included that PAP could be gamified or modelled on existing “get started” mobile apps to better attract young users. A PAP mobile app could also be a better tool to continuously provide support and help boost motivation in the child and parent. Participants expressed that this would be a way to “enter” the digital world that many children are used to these days. Visual aids were perceived as an important facilitator for communicating with both child and parent, to support their work with PAP.

### 3.3 Collective action: Collaboration around PAP across settings

In relation to this construct, the participants described their experiences of working together to enact PAP and both managers and staff agreed that they face multilevel challenges and complexities when working with PAP. The participants experienced facilitators and barriers when they compared their different organisational and contextual conditions.

#### 3.3.1 Organisers of physical activities playing a vital part

The organisers of physical activities received much attention from the participants. They expressed concerns related to *interactional workability;* not only that it is difficult to know the current range of activity offerings, but also that the offerings vary depending on whether the child lives in Gothenburg or one of the surrounding municipalities. This was considered unequal by several managers, and affecting how the staff are able to work with PAP. A perceived barrier for working with PAP was that the Gothenburg activity catalogue is limited to the city and not surrounding municipalities. This limitation is an example of inadequate organisational support, thus affecting *contextual integration* of PAP.

Participants agreed that PAP entitles the child to cost-reduced activities, and they used the written prescription to send children to activity organisers and to signal that the child may need extra support to get started. A problem pertaining to *relational integration*, formulated by managers, was a lack of feedback about how the child fared or whether the activity was completed. Managers were also concerned about the activity organisers' competence in supporting children with obesity, who were perceived to differ from children without obesity, e.g., by having motor difficulties, being unaccustomed to movement, maybe being ashamed of their bodies, or having a neuropsychiatric diagnosis. Participants suggested adapting exercise to get started and supporting the children to find their place and not get lost in the association life.

*There are those who absolutely do not want to exercise because they are ashamed of their bodies.* (A19, physician)

*The difficult thing is those who don't fit into independent gym training. When they go to a gym, they find it difficult that there is no one who looks like them. So, you feel that you don't fit in the gym.* (A20, physiotherapist)

*I also think about your dance //…//. It's incredibly popular and highly appreciated. It's fantastic because it's a type of movement that everyone can do according to their own conditions.* (A21, nurse)

#### 3.3.2 Complex family situations

The participants agreed that the children often live in complex family situations affecting the *interactional workability* with PAP; in particular socioeconomic vulnerability, cultural differences, and parental conditions were perceived as such. Their experience was that parents might not mention that poor finances prevented them to help their children get started with an activity.

*I feel that many people are happy when they receive it [PAP]. It's like a little gift. //…// It's something you give them so they can get started.* (A18, nurse)

*Our children cannot afford to go to activities even though they want to. It's an area with low economic status and it's also a feeling that they want to get it a little cheaper. For me it was an opportunity to stimulate them to physical activity on the condition that they can manage it financially.* (A19, physician)

The participants perceived that parents work a lot and have limited time to sign up their child, transport them, or participate themselves in activities. In large families, they experienced that resources need to be distributed amongst siblings, which might affect participation for an individual child. They also experienced that parents living in unsafe areas avoid letting their children outside in fear of undesirable events.

For families with limited participation in society at large, cultural differences must be considered, according to the staff. Two key barriers were perceived to be language difficulties and families being unaccustomed to outdoor activities and equipped for different weather conditions, limiting family-oriented activities.

Participants had experiences of parents suffering from lifestyle-related health issues, such as overweight, obesity, or type 2 diabetes. They had had both a parent and a child in PAP treatment together, with positive results for the parent. On the other hand, participants also experienced that parents were ashamed to come, entailing a risk of losing contact with the family so that the child's treatment could not be completed.

Taken together, all these complexities in family situations had consequences for what types of PA the participants could offer to the child and on their ability to work with PAP, thereby affecting *interactional workability* of the method.

#### 3.3.3 Using a family-centred approach

Staff described their general work in the paediatric clinic as being family-centred, albeit with particular focus on the child. As most staff were trained in person-centredness or family-centredness, *skill-set workability* could be seen as adequate in this regard. The participants agreed that the family-centred and individually tailored approach in working with PAP makes much sense for these families. When using PAP, the participants experienced that families were involved to a high extent in setting goals and finding joyful activities for the child. For the child, it is often about finding inner motivation without focusing on their weight. Additionally, both staff and managers strongly agreed that the families need well-coordinated support in the transition between clinics or physical activity organisers, so that the family-centredness would not get lost—as is often the case. This might manifest itself as a delay in the onset of the treatment, or that the individually tailored prescription is not picked up by the recipient.

A shared experience by the participants was working with what they called “family PAP”. It was described as a family treatment to facilitate getting started together, where the parents could also set their own goals.

*I miss the opportunity to write PAP to parents too. A concrete tool would be that you can work with the whole family. It's quite often that you get questions from parents as well, what to do //…//.* (A18, nurse)

*Yes, I think it's extremely common that the parents are not physically active either. //…// There are quite a few parents who are nevertheless positive about getting some activity done and understand that it's good to exercise.* (A20, physiotherapist)

### 3.4 Reflexive monitoring: new insights about PAP

The participants continuously evaluated and reevaluated their understanding of working with PAP and agreed to how it could change, based on their own experience. The expressed desire for clear guidelines on how to work with PAP reflected a need for *systematisation* in terms of easier access to research reports about effects of PAP.

#### 3.4.1 Participants reconfiguring their work after receiving information about the method

The participants were briefly informed about the components of PAP during the focus group discussions, and some figured, after they had been informed, that they could work in a different way based on both the clinic's *communal appraisal* and their *individual appraisal* of PAP. They perceived that the work could be more person- and family-centred and that it should focus more on everyday activities and activities for the family, instead of only writing a prescription to an activity organiser.

*My traditional PAP has just been the written prescription based on the activity catalogue. It feels very positive to hear that it counts as PAP, this so-called individual conversation where you make an assessment and an agreement that feels reasonable //…//. Then follow up.* (A16, physician)

*I haven't really thought about it like that, that you can start by writing a prescription for a suitable everyday activity. That it will be like a small contract between us. //…// I'm going to take that with me and do something like that.* (A17, nurse)

#### 3.4.2 Determinants for working with PAP exist in all domains

The participants reflected on different factors that affected their work with PAP. While the method was experienced as a helpful tool for working with PA change, the varying understanding of PAP and its components was perceived as making a common understanding of how to work with PAP challenging. A lack of clinical guidelines and a lack of physiotherapists in paediatric healthcare were hindering factors for working together to enact PAP, as was the actual prescription and the limited knowledge and availability of activity offerings. The involvement of physiotherapists, either as adjunct to paediatric healthcare or in a rehabilitation setting, and better information and availability of activity offerings were discussed as important facilitators for working with PAP. The barriers and facilitators that came up during the discussions, based on the participants' experiences and expectations of PAP, are summarised in Table 4. Most determinants were related to cognitive participation and collective action, constructs that require collaboration and enacting of PAP.

**Table 4:** Determinants for working with physical activity on prescription for children with obesity, perceived by staff and managers.

Barriers	Facilitators
Coherence	
Using PAP to lose weight Differentiating PAP from usual ways of working Uncertainty of PAP and its components	A tool for working with PA and sedentary behaviour
Cognitive participation	
Lack of formal guidelines for the work with PAP	A concept for how to work with PAP
Physiotherapists working outside paediatric health care	Physiotherapists working with PAP Physiotherapists coming to the paediatric clinics facilitate transitioning between clinics
The written prescription is used as a classic medical referral	
The written prescription is time consuming and non-practical	
Limited knowledge of current activity offerings, including information	Increased availability of local activities and information through a web-based compilation
	Digitising PAP including an activity catalogue
Collective action	
Activity offerings vary depending on where the child lives	PAP justifies cost-reduced activities
The activity catalogue not available outside the city	Availability of and information about local activities in regional areas
Lack of feedback about the child from PA organisers	Adapted training for the children to get started and support to find their place
Lack of knowledge of PA organisers’ competence around children with obesity	
Socio-economic vulnerability in the family Cultural differences Parental ill-health	Family-centred approach
Lack of support for families in the transition between clinics or PA organisers	Support for families in the transition between clinics or PA organisers
Reflexive monitoring	
Misconceptions of the PAP components	Re-evaluation of working method

PAP, physical activity on prescription; PA, physical activity.

## 4 Discussion

This focus group study explored experiences of working with PAP for children with obesity amongst twenty-six healthcare professionals and managers in paediatric outpatient clinics in western Sweden. The findings help explain how the clinics work with PAP and why the work differs amongst the different clinics. Key findings are that the PAP work was described as non-uniform, and that there is a perceived need to enhance the understanding of the PAP components and develop guidelines for PAP work for children. Another key finding is that the work with PAP for children with obesity was perceived as a method to help the children get started with PA and not for them to lose weight. The children were perceived to have great needs, and collaboration with physiotherapists and PA organisers was considered necessary, but the participants pointed at several barriers that need to be addressed to better meet the children's needs. In the transition between clinic and PA organiser, the participants experienced that the family-centred approach, considered central for this patient group, was lost.

The work with PAP was mainly perceived as helping children to get started with PA, rather than to lose weight. This view is consistent with previous research in adults, in which PAP according to the Swedish model has yielded significant increases in PA (22) while effects on secondary outcomes, such as body weight and waist circumference, have been negligible (22). Although these findings may not be applicable to the child population, they support the finding in our study of what the perceived main benefit of the intervention is. To avoid misunderstandings amongst the families about the purpose of PAP for children, the participants emphasised the importance of providing clear and accurate information to facilitate communication and avoid stigmatisation amongst parents and children.

Another purpose of PAP that was not as clearly stated by the participants, was to decrease sedentary behaviour, possibly because this was considered a challenging and new goal. According to previous research, the overall trend of increased screen time amongst young people is a great health concern due to its association with adiposity and cardiometabolic risks (42). Parents need support to model healthy screen behaviours for their children, while children need encouragement to limit their use of devices in exchange for social interaction and outdoor play (43). As an approach to movement behaviour, the European Childhood Obesity Group suggests using anti-obesity strategies that target both PA and sedentary behaviour (44), indicating that PAP for children with obesity should include both strategies. Empowering staff to increase their confidence in addressing sedentary behaviour may further enable such work.

Differentiating an intervention from usual ways of working is an important prerequisite for implementation (36). However, it was unclear how the participants distinguished the PAP components from their usual clinical practice, resulting in some confusion. For example, some participants handled the written prescription in PAP like a medical referral, where the recipient is expected to return a response when the assignment on the referral is completed. The use of PAP includes a follow-up responsibility, which should be clarified at the clinics.

Another example was that although PAP could be used at the paediatric clinic without the prescription being sent to either another healthcare clinic or a PA organiser, this was a less common way of working and not known by all participants. When it occurred, the written prescription was seldom used, as it was considered both time-consuming and impractical—a finding that is consistent with other studies (29, 30). Instead of using PAP, including the written prescription at the clinics, the participants preferred to rely on regular practice routines for working with PA. However, some participants reflected about the benefits of PAP compared to their regular routines, and difficulties in differentiating methods from each other are not unusual (36). The fact that PAP was originally not designed for paediatric healthcare (28), and excessive use of the word PAP in unjustified contexts, might also contribute to misconceptions and disadvantage the intervention.

On the other hand, participants working with motivation and change of PA habits could clearly define the core components of PAP and found them useful, especially the person-centred dialogue component. This finding is in agreement with a recent study amongst school nurses, who expressed that working with PAP facilitates dialogue about the child's motivation and selection of PA (25). The results of the present study indicate that PAP could be more meaningful and clearer for paediatric professionals, who may also have to overcome a certain knowledge gap. This knowledge barrier has been identified in previous studies (28, 30).

The managers agreed that there is a lack of structure for how to work with PAP and how the staff work on finding practical solutions. Despite the unclear structures, which are well documented and reported (29–31, 45), paediatric practitioners have recently shown receptivity to using PAP for children with obesity (28). Although it could be interpreted as good micro- and meso-level prerequisites, there is still a need for macro-level change. Since implementation is a multilevel activity, changes are required at different levels within and outside the healthcare system (46). Without clear guidelines, healthcare services cannot be distributed evenly amongst citizens (45), which may exclude the possibility for children with obesity to participate in PAP. However, guidelines are based on currently available research and since there is little research on PAP for children, a basis for such is primarily needed. Physiotherapists were described as one of the most important professions in the work with PAP and were also perceived to have an important collaborative role. Their competence was regarded as necessary for many children who were considered to have psychomotor needs. Likewise, the most common problem reported by parents regarding their children is gross and/or fine-motor skill dysfunction, a finding that has been shown to be five times more than the expected rate for children with obesity (47). The physiotherapist's expected involvement in PAP is thus well justified and consistent with the research (24, 30, 48, 49). Recently, physiotherapists together with nurses have reported PAP to be a normal part of their work for children with obesity compared to other professions (28). This finding indicates the inclusion of physiotherapists in the delivery of PAP for children with obesity in collaboration with paediatric clinics, although the conditions for collaboration may vary.

The other collaborators brought up by participants were PA organisers to whom children and their families were sent, to participate in PA. Although PAP often entitles price-reduced PA, a huge barrier was perceived to be the inconsistent premises for collaboration between paediatric healthcare and PA organisers. This included everything from the handling of the prescription, lack of information about the range of activities and its content to uncertainty about the competence of the PA organisers in childhood obesity. Since the diagnosis is not infrequently accompanied by neurodevelopmental disorder, deviant motor skills (47, 50) and social vulnerability, an approach adapted to the children and their families is highly warranted. This is in accordance with the findings of a report from the Public Health Agency of Sweden (45), which identified a lack of evidence for PAP for children and limited collaboration with PA organisers. Although PAP justifies reduced activity costs, which is helpful to parents who are financially challenged, the results of the present study show that this is not sufficient. The participants perceived difficulties in assisting the children and their families into organised sports activities, where structures for collaboration are inconsistent or missing. Another barrier perceived by the study participants was that PAP is time consuming and non-practical, which is in line with the findings of a previous study in which lack of time and resources for working with PAP for children with intellectual disability were described by paediatric healthcare practitioners (32). However, the finding of lack of experience of PAP that was described for the work with PAP for children with intellectual disability was not in accordance with the present study where the participants perceived themselves as having experience of working with PAP.

PAP for children is intended to be used in conjunction with a family-centred approach (20), which is widely used in paediatric settings and is believed to be the most effective way to provide care for children (51). The participants were not satisfied with how the family-centred care was adhered to in the work with PAP and perceived that the intervention deviated from regular practice routine. The reasons were foremost the unclear premises for collaboration and referral pathways causing treatment delays and communication failures. The need to coordinate care throughout the intervention (30, 52) and treatment delay (52) have also been reported by professionals working with PAP for adults. Especially when working with PA and PAP for children, collaboration with stakeholders is crucial (23, 25, 53). However, their concern was mainly related to outsourced PAP treatment and less pronounced for PAP work in the paediatric clinics. Here, the initial goal could be to increase everyday PA with the family, reduce sedentary behaviour, and, in a second step, engage the child and family in organised sports activities. To better align with the family-centred approach, this could be a strategy when introducing PAP, which may better suit families with complex life situations, when collaboration premises are not optional. One of the findings of this study highlight the collaboration with PA organisers, which must be further developed if the family-centred approach is to be maintained when using PAP in paediatric healthcare.

Although the family-centred approach was considered fundamental, another more loosely assembled proposal, involving the family even more, emerged. The participants called it “family-PAP” and the objective could be to involve the family in goalsetting for example joint family/child goals or both child and parent goals. Research has shown that including parents and family is superior to treatment of children alone (54) and one family-focused intervention that yielded improvements in sedentary behaviour and total PA suggested that parents' PA habits are mediators of PA engagement in young children (55). The authors proposed including siblings and parents of varying physical abilities, minimising competitive moments, minimising necessary equipment, and accounting for weather variations. Although behavioural-changing interventions for children with obesity should preferably be family-based (56), there is an overall lack of family-based approaches to improve PA in children (57). Further research on PAP for children with obesity is highly desired, both in terms of intervention effectiveness and from an implementation perspective. Use of PAP in other contexts, such as rehabilitation clinics and amongst activity organisers, should be investigated. To capture the patient perspective, research on experiences of children and their families of participating in PAP is also highly warranted.

Many of the identified determinants for working with PAP could be addressed in developing clinical guidelines. A guideline would in itself address the expressed barrier that a guideline for treating children with obesity with PAP is lacking. It would be important to address ambiguities and improve clarity related to terminology, definitions, purpose, and content of PAP and its components. The guideline should be cross-disciplinary and include organisational aspects and pathways, to improve collaboration within and outside paediatric healthcare. Adjunct to a guideline, information about available physical activities should be improved, for example by digitising activity catalogues.

### 4.1 Methodological considerations

The chosen focus group methodology contributed to a deeper understanding of how this sample of staff and managers in paediatric healthcare experience the work with PAP for children with obesity. The method was considered a strength as it enabled us to capture multiple perspectives deriving from the interaction amongst participants, essential to understand the complex nature of PAP work in relation to its implementation prerequisites. Using NPT as an analytical framework helped explain the experiences of working with PAP, and the core constructs and subconstructs helped interpretation at a deeper level. Applying NPT in the deductive analysis phase allowed us to focus on the collective experience of working with PAP. Nevertheless, it was sometimes challenging to characterise and assign findings and categories to the different themes and NPT constructs, due to the themes being interrelated and the overlap between the constructs. This overlap has been acknowledged in previous research (58).

Using a sample of staff and managers who varied in age, work location, professions, and experience of working with PAP ensured that a variety of descriptions were obtained. The researchers represented two different professions (physiotherapists and nurse), and one was working in a different regional area than the others, which could be an advantage during the analysis process and strengthen trustworthiness. To strengthen credibility and dependability the study procedures have been described in as much detail as possible and during the analysis the research team was continuously collaborating, which was also a way to handle preunderstanding.

There were some limitations to the study. The study was conducted in one healthcare region in Sweden, and findings may not be applicable to other healthcare regions or other countries, in which work in paediatric clinics may be organised differently. Although confidentiality was emphasised, it was difficult to prevent participants from discussing the study at the paediatric clinics, which may have affected the participants' expectations. This is a common problem in focus groups connected to organisations (33). In one of the groups consisting of staff, one manager participated which may have caused a power imbalance within the group.

## 5 Conclusions

Physical activity on prescription is a well-known intervention that is inconsistently used for children with obesity. It is primarily used to help the children get started with physical activity rather than to achieve weight reduction. However, the PAP work is experienced as unclear, and needs to be better structured. Establishing a common terminology related to PAP could enhance comprehension of its components. Collaboration between physiotherapists, physical activity organisers, and healthcare professionals is essential to meet the children's needs and reduce transitions, ultimately promoting a more consistent and effective use of PAP.

Further research is needed to confirm our findings, refine the PAP methodology, and develop guidance for its application in management of childhood obesity. The intervention should include a family-centred approach for this patient group. PAP also needs to align better with existing collaborations with other healthcare units as well as with new forms of collaboration with physical activity organisers in the community.

## Data Availability

The raw data supporting the conclusions of this article will be made available by the authors, on reasonable request.
