# Supplementary material for: Physical activity on prescription for children with obesity: a focus group study exploring experiences in paediatric healthcare
Source: Front Health Serv. 2024 Apr 4;4:1306461. doi: 10.3389/frhs.2024.1306461 (PMC11024476; doi:10.3389/frhs.2024.1306461)
Supplement: Supplementary file 2 [file Table2.docx]

**Discussion guide**

|  | **Topic** | **Prompt** |
| --- | --- | --- |
| **Icebreaker** | What is PAP, Physical activity on Prescription? | What does PAP mean to you?  *Summarise* |
| **Key questions** | 1. What are your experiences/expectations of working with PAP for children with obesity?        2. How is work with PAP affected by the families’ conditions?    3. What do you think about the organisational prerequisites in your clinics for working with PAP?    4. How do you experience the collaboration with activity organisers? | What do you experience works well?  What do you experience works less well?  In what way does PAP differ from other ways of working with physical activity?  How are PAP issues managed at your workplace?  -individually/group…  -a common goal…  *Summarise*    What would you do if a parent or a colleague requests PAP treatment?  -Do you see any effect of PAP?  -social, cultural, financial…  -motivation, lifestyle  -What is it like to work with parent support?  *Summarise*  Support from managers:  -time, resources…  -training…  What are your needs?  *Summarise*  Collaborations outside health care…  -Can you give examples?  *Summarise* |
| **Rounding off** | If you were to give us some advice on how to adapt PAP, what would it be?    Is there anything else that you want to talk about, that hasn’t already been discussed? | *Summarise*      Can we contact you afterwards in case we need any clarifications? |
